# Bacteriospermia and its antimicrobial resistance in relation to boar sperm quality during short-term storage with or without antibiotics in a tropical environment

**DOI:** 10.1186/s40813-023-00320-2

**Published:** 2023-05-17

**Authors:** CongBang Ngo, Junpen Suwimonteerabutr, Nuvee Prapasarakul, Jane M. Morrell, Padet Tummaruk

**Affiliations:** 1 Department of Obstetrics, Gynaecology and Reproduction, Faculty of Veterinary Science, Chulalongkorn University, Bangkok, 10330 Thailand; 2 Department of Veterinary Microbiology, Faculty of Veterinary Science, Chulalongkorn University, Bangkok, 10330 Thailand; 3 Department of Clinical Sciences, Swedish University of Agricultural Sciences, Uppsala, 75007 Sweden; 4 Center of Excellence in Swine Reproduction, Chulalongkorn University, Bangkok, 10330 Thailand; 5 Center of Excellence in Diagnosis and Monitoring for Animal Pathogens, Chulalongkorn University, Bangkok, 10330 Thailand

**Keywords:** Acrosome integrity, Antibiotics, Boar, Chilled semen, Seminal bacteria

## Abstract

**Background:**

In tropical environments, boar semen is prepared either from a boar on the same farm as the sow herd or collected in semen collection centers and then transported to other farms. Thus, the semen doses can be used for artificial insemination either immediately or preserved for 2–3 days. The present study investigated the bacteriospermia and its antimicrobial resistance in relation to boar sperm quality during short-term storage in semen extender with or without antibiotics in Thailand.

**M&M:**

In total, 20 Duroc ejaculates were collected. Each ejaculate was diluted in Beltsville Thawing Solution extender either with 0.25 g of gentamicin per liter (ANTIBIOTIC) or without gentamicin (NO-ANITIBIOTIC) to create semen doses containing 3,000 × 10^6^ sperm/100 mL. These were stored at 17 °C for 4 days. Semen characteristics and total bacterial count (CFU per mL, log_10_) were measured after collection and during storage.

**Results:**

Sperm viability was decreased by 6.4% for every 1.0 log_10_ increase in total bacterial count (*p* = 0.026) and *Staphylococcus spp.* were the most frequently isolated across ejaculates. Throughout the 4 days of storage, sperm motility, viability and acrosome integrity in the ANTIBIOTIC group were higher than those in the NO-ANTIBIOTIC group (*p* < 0.05), while the total bacterial count was lower (1.9 ± 0.1 versus 3.9 ± 0.1 log_10_, respectively; *p* < 0.001). Without antibiotic supplementation, the total numbers of bacteria counted on days 2 and 3 of storage were higher than those determined on days 0 and 1 (*p* < 0.001). Differences in semen quality were detected on days 2 and 3 between the NO-ANTIBIOTIC and ANTIBIOTIC groups in high-viability semen (*p* < 0.05). However, no differences in sperm quality between the NO-ANTIBIOTIC and ANTIBIOTIC groups were detected in the low-viability semen on each storage day (*p* > 0.05). On the last day of preservation, *Globicatella sanguinis (*57.2%), *Delftia acidovorans (18.9%)* and *Micrococcus spp.* (5.9%) remained as the top three most abundant contaminants in the semen with antibiotic.

**Conclusion:**

Our findings contribute new insights toward reducing antibiotics as well as rational antibiotic use in the boar AI industry. The growth of bacteria was significantly greater only after 2 days of preservation in the semen without antibiotic. For semen doses diluted from highly viable ejaculates, it is possible to store for 2 days without any antibiotic supplementation. Moreover, bacterial counts increased at the end of storage in the presence of gentamycin, suggesting the loss of bacteriostatic properties of gentamicin to the growth of bacteria during storage.

## Background

Bacterial contamination during boar semen collection and subsequent processing is unavoidable [1, 2]. At the stud/farm, sources of bacterial contamination can be classified into being either of animal origin, such as faeces, preputial cavity fluids, skin, hair and respiratory secretions from boars, or of non-animal origin, such as water, feed, ventilation system, collection area, and lab-air handling system [3, 4]. There are two or three bacterial contaminants in most ejaculates in Poland [5], with *Staphylococcus*, *Streptococcus* and *Pseudomonas* being the most frequently isolated bacterial genera. In addition, *Enterobacter*, *Bacillus*, *Proteus* and *Escherichia coli* were also detected in boar semen [5]. In Cuba, 75% of the ejaculates are contaminated with at least one bacterial type, with *Escherichia coli* being the most common contaminant. Other bacterial genera, including *Proteus*, *Serratia*, *Enterobacter*, *Klebsiella*, *Staphylococcus*, *Streptococcus* and *Pseudomonas* were also detected [6]. Recently, Wang et al. [7] found that *Streptococcus porcinus* isolated from vaginal secretions of sows with endometritis originated from infection during artificial insemination (AI) and was resistant to aminoglycosides and tetracyclines. In tropical environment, common microbes causing endometritis, abnormal vaginal discharge and return estrous after AI include *E. coli*, *Streptococcus sp.* and *Staphylococcus sp.* [8]. In most of the swine herds in Thailand, semen doses used for AI are generally prepared within the herds or in a boar stud close to the sow herd [9]. Thus, the semen can be used either immediately or preserved for 2–3 days. However, comprehensive studies on bacterospermia and the evidence of antimicrobial resistance to the commonly used antibiotics in relation to semen quality during short-term storage in Thailand are scarce.

Although bacterial contamination differs among specific areas, such as countries or artificial insemination centres, the presence of bacteria in semen has a universal detrimental effect on sperm quality [10]. Bacteria and sperm compete for nutrients in semen extender; metabolic byproducts from live bacteria and lipopolysaccharides released from the cell walls of dead bacteria can be harmful to spermatozoa [11]. In chilled extended semen, the total bacterial count increases during storage, and disruption of the sperm plasma membrane and acrosome integrity also increase [5]. Another study reported a negative correlation between bacterial contamination and sperm parameters such as sperm motility and viability [12].

The rational use of antibiotics in semen extenders and the replacement of conventional antibiotics in extenders by alternatives are two main directions to tackle unavoidable bacterial contamination and its harmful effects on semen quality, and to minimise global antimicrobial resistance threats [13, 14]. Resistance was observed against antibiotics commonly used in commercial porcine semen extenders [15]. Thus, taking control of antimicrobial resistance is a fundamental requirement for a sustainable pig breeding industry [13]. Alternatives to antibiotics and a gradual change in antibiotic use are a consequence of the antibiotic resistance observed among isolates from boar semen. Alternatives could be the use of antimicrobial peptides, which are synthesised in mammalian organisms e.g., epithelial tissue, the digestive tract or the reproductive tract [16, 17], the separation of sperm from bacteria using physical methods such as single-layer centrifugation with low density colloid [18–20], hypothermic preservation around 5 °C [21] and the use of miscellaneous substances such as iodine methionine [22]. During the last few decades, a combination of penicillin and streptomycin was commonly used in most boar semen extenders due to limitations in the choice of available antimicrobials compatible with sperm survival [3, 23]. However, the updated international guidelines for the prudent use of antibiotics in boar semen extenders indicate that beta-lactam antibiotics such as penicillins and cephalosporins, as well as extenders with cocktails or undeclared antibiotic contents, are not allowed [13, 24].

Although antibiotic replacement methods have shown their advantages in eliminating or effectively controlling bacterial growth during semen preservation [19], the rational use of antibiotics and applying strict hygienic measures during collection and laboratory processes are preferable in pork-producing countries because of the high costs of antimicrobial peptides [25]. Moreover, semen preparation techniques related to physical bacterial elimination methods require skilled technicians, which increases the semen processing time and may decrease the quality of semen doses [26]. Broad-spectrum aminoglycosides, such as gentamicin, satisfy the regulations proposed by breeding organizations worldwide regarding boar semen trade in the global market; they are also the most commonly used antibiotics [13, 27, 28]. Generally, commercial semen extenders, such as Beltsville Thawing Solution (BTS) and Androstar Premium, are supplemented with 0.25 g of gentamicin per litre [1]. At this concentration, gentamicin prevents the spread of diseases and sperm degradation while minimizing the threat of antibiotic resistance [28]. Although the use of gentamicin is common and satisfies global regulations, available data regarding its bacteriostatic effects in boar semen samples of various quality are lacking. It is possible, for example, that addition of antibiotics might impair sperm quality in poor quality semen even further. The present study therefore investigated the effects of bacteriospermia on boar semen quality during short-term preservation with and without antibiotics.

## Materials and methods

### Animals

The study was carried out in a boar stud located in the western part of Thailand. In total, 20 ejaculates were collected from 20 Duroc boars. All boars were proven sires and aged between 1 and 3 years. The boars were kept in individual pens (9 m^2^ per boar) in a closed house equipped with an evaporative cooling system. Each boar was fed 2.5–3.2 kg of commercial feed daily as a standard diet, and water was provided ad libitum via water nipples. The experiment was conducted from December 2021 to January 2022.

### Experimental design

For the 20 Duroc boars included in the experiment, each ejaculate was split and diluted in two types of semen extender: Beltsville Thawing Solution (BTS) without gentamicin (NO-ANTIBIOTIC; n = 20) and BTS with 0.25 g/L of gentamicin (ANTIBIOTIC; n = 20) to produce semen doses containing 3 billion spermatozoa in 100 mL. The diluted semen was preserved for 4 days, including the semen collection day (day 0) and the first, second and third days of preservation (i.e., days 1, 2 and 3, respectively). Sperm quality parameters including semen volume, sperm motility, sperm viability, sperm concentration, and pH of fresh semen were measured immediately within 15 min of semen collection. During storage, sperm motility, viability, acrosome integrity, sperm membrane permeability and mitochondrial activity were evaluated starting at 11:00 h of every storage day. Bacterial culture was performed at 11:00 h to determine the total bacterial count (CFU/mL, log_10_) and specific isolated contaminants in fresh and diluted semen.

### Semen collection and processing

The semen was collected between 06:00 and 07:00 h, using the gloved-hand method. An ejaculate was collected from each boar every 5 to 7 days. Immediately after collection, semen volume (50–300 mL) and pH were measured. Sperm concentration (range of 50–600 × 10^6^ sperm per mL) was evaluated using a Spermacue® (Minitube, Tiefenbach, Germany). The total sperm per ejaculate was calculated by multiplying semen volume by sperm concentration. Subjective sperm motility and viability were evaluated microscopically at 200× magnification by the same technician. Semen samples with a sperm motility of ≥ 65% were diluted in sterile BTS with and without gentamicin to produce semen doses. The ingredients of the BTS semen extender were 205 mM glucose (C_6_H_12_O_6_), 20.4 mM sodium citrate (Na_3_C_6_H5O_7_), 10.0 mM potassium chloride (KCl), 15 mM sodium bicarbonate (NaHCO_3_) and 3.36 mM ethylenediaminetetraacetic acid disodium salt dihydrate (EDTA); the pH was adjusted to 7.2 [1].

### Semen evaluation

Total sperm motility and the motion characteristics including straight-line velocity (VSL, µm/sec), curvilinear velocity (VCL, µm/sec) and average path velocity (VAP, µm/sec) were evaluated using a computer-assisted sperm analysis system (SCA® CASA System, MICROPTIC S.L., Barcelona, Spain). The CASA system was set for boar sperm and applied with a frame rate of 50 frame per second and box size of 100 pixels. The minimum and maximum area for the objects were 10 and 80 µm^2^, respectively. The motile spermatozoa were set for static (< 10 μm/s), slow-medium (< 25 μm/s), and progressive motility (> 45 μm/s). The diluted semen was placed in a chamber and examined on a warmed stage (TOKAI HIT, Shizuoka-ken, Japan) at 37 °C under a phase-contrast microscope (BX41, Olympus, Shinjuku, Japan). The proportion of motile sperm was quantified from 1500 sperm cells in five randomly selected fields for each sample [29].

Sperm viability was determined using SYBR-14/EthD-1 (Fertilight®, Sperm Viability Kit, Molecular Probes Europe, Leiden, the Netherlands). A 10-µL aliquot of diluted semen sample was mixed with 1 µL of 14-µM EthD-1 (Molecular Probes Inc., OR, USA) in 1 mL PBS and 2.7 µL of 0.38-µM SYBR-14 (Dead/Alive Kit; Molecular Probes Inc.) in 1 mL dimethyl sulfoxide (DMSO) and then incubated at 37℃ for 15 min. Finally, 200 sperm cells were examined using a fluorescence microscope (CX-31; Olympus, Tokyo, Japan) (1000X). Sperm cells stained only green were classified as live, with an intact plasma membrane. Sperm cells stained only red or both green and red were considered to be dead or have damaged plasma membranes, respectively. Sperm viability was calculated according to the proportion of live sperm with an intact plasma membrane [30].

Acrosome integrity was measured using EthD-1 (Fertilight®, Sperm Viability Kit, Molecular Probes Europe, Leiden, Netherlands) and fluorescein isothiocyanate-labelled peanut (Arachis hypogaea) agglutinin (FITC-PNA) staining (Sigma-Aldrich Co. Ltd., St Louis, MO, USA). A 10-µL aliquot of the diluted semen sample was mixed and incubated at 37 °C for 15 min with 10 µL of 14-µM EthD-1 (Molecular Probes Inc., OR, USA). After incubation, one 8µL drop of the mixture was smeared on a slide and air-dried at room temperature. The dried slide was dipped into 95% ethanol for 30 s. before staining with 15 µL FITC-PNA solution [FITC-PNA in PBS (1:10, v/v)] at 4 °C for 30 min in a moist chamber, followed by washing in cold PBS solution at 4 °C and air-drying at room temperature. The acrosome status of 200 sperm per sample was evaluated by fluorescence microscopy (CX-31; Olympus, Tokyo, Japan) (1000X). The proportion of sperm with an intact acrosome, i.e., with an acrosome cap stained green (positive), was calculated. Sperm cells stained orange and without an acrosome cap, with a green band at the equatorial segment or with a disrupted patch-like appearance of the acrosome cap were classified as sperm with acrosome damage [30].

Sperm plasma membrane permeability was evaluated using the short hypo-osmotic swelling test (sHOST). A 10-µL diluted semen sample was mixed with 200 µL of citrate buffer (75 mOsM) and then incubated in the dark at 37 °C for 30 min in a 1.5-mL Eppendorf tube. After incubation, 175 µL of HOS solution with 5% formaldehyde (75 mOsM) was added. Subsequently, an 8 µL drop of semen sample was placed on a glass slide. The appearance of the tails of 200 sperm cells was evaluated by light microscopy (400X) and classified as positive (sperm with a coiled tail) or negative (sperm with a straight tail). The proportion of positive sperm indicates a functional sperm membrane [9, 30].

Mitochondrial activity was accessed using the fluorochrome 5,5′,6,6′-tetrachloro-1,1′,3,3′-tetraethylbenzimidazoly-carbocyanine iodide (JC-1; Molecular Probes, Molecular Probes Inc., Eugene, OR). First, 12.5 µL of diluted semen were mixed with 25 µL of JC-1 solution composed of 1.6 µL of 0.153 mM JC-1, 1 µL of 0.02 mM SYBR-14, 1.6 µL of 2.4 mM PI in 100 µL HEPES-buffered medium, followed by incubation at 37 °C for 30 min. Finally, one drop of 8 µL of stained semen sample was placed on a glass slide, and 200 sperm cells were evaluated using a fluorescent microscope (CX-31; Olympus, Tokyo, Japan) (1000X). Sperm with yellow-orange fluorescence at the midpiece were classified as positive (high mitochondrial membrane potential), whereas spermatozoa with less green or no green fluorescent colour at the midpiece were classified as negative (low mitochondrial membrane potential) [9, 30].

### Bacterial evaluation

Total aerobic contaminants and isolated bacteria in both fresh and extended semen were quantified by bacterial culture on blood sheep agar [5, 6]. Briefly, 1 mL of the fresh and extended semen samples was diluted in tubes containing 9.0 mL of PBS (0.1 M phosphate buffer containing 0.15 M NaCl, pH 7.3) to prepare serial dilutions (10^0^ – 10^6^). From each dilution, 1.5 mL was plated on three agar plates (0.5 mL/plate) and incubated aerobically at 37 °C for 24 h. Plates with fewer than 300 CFU/mL were selected for bacterial counts. The total bacterial count was calculated as the average number of colonies on the three different plates; this number was then subjected to logarithmic transformation (CFU/mL, log_10_). Subsequently, matrix-assisted laser desorption ionisation-time of flight mass spectrometry (MALDI-TOF MS) (Microflex® LT, MALDI BiotyperTM System, Bruker, Germany) was used for bacterial identification. The colonies that grew after 24 h of incubation at 37 °C were selected based on their morphological and haemolysis characteristics. The bacteria were then subjected to a preparatory extraction with 1 µL of formic acid (FA) and 1 µL of alpha hydroxyl l,4 cinnamic acid matrix (HCCA) and finally analysed in a Bruker Biotyper MALDI-TOF [31, 32].

### Statistical analysis

The statistical analyses were carried out using the SAS statistical software version 9.4 (SAS Inst. Inc., Cary, NC, USA). Shapiro-Wilk test and Qualitative-Quantitative plots (Q-Q plots) were used for testing the normal distribution of the continuous data. Boar sperm characteristics and bacterial count in semen were analysed using the general linear model procedure of SAS. Spearman correlation and linear regression were applied to analyse the association between sperm parameters and bacterial contamination in diluted semen samples. Semen evaluation data including semen volume, pH, sperm concentration, total number of sperm per ejaculate, sperm motility, sperm viability, acrosome integrity, sperm plasma membrane function, mitochondrial activity, number of identified bacteria and average total bacterial count were analysed by multiple analysis of variance, using the mixed model procedure of SAS. The total bacterial counts were log_10_-transformed before statistical analysis. The statistical models included the fixed effect of treatment groups (with and without antibiotic), storage day (days 0, 1, 2 and 3) and two-way interaction. Boar identity was included in the model as a random effect to adjust for repeated measurement. Least-square means were obtained from each class of the factors and compared using the least-significant difference test. In addition, the sperm viability among ejaculates were ranked from lowest to highest (1 to 20) and the analyses on the effect of the antibiotic on the boar semen quality were also carried out in the low viability semen (mean = 69.6 ± 1.5%, n = 10) and high viability semen (mean = 83.8 ± 1.5%, n = 10). The differences with *p* < 0.05 were considered statistically significant.

## Results

### Sperm quality evaluation and bacterial culture in fresh semen

**Fig. 1:**
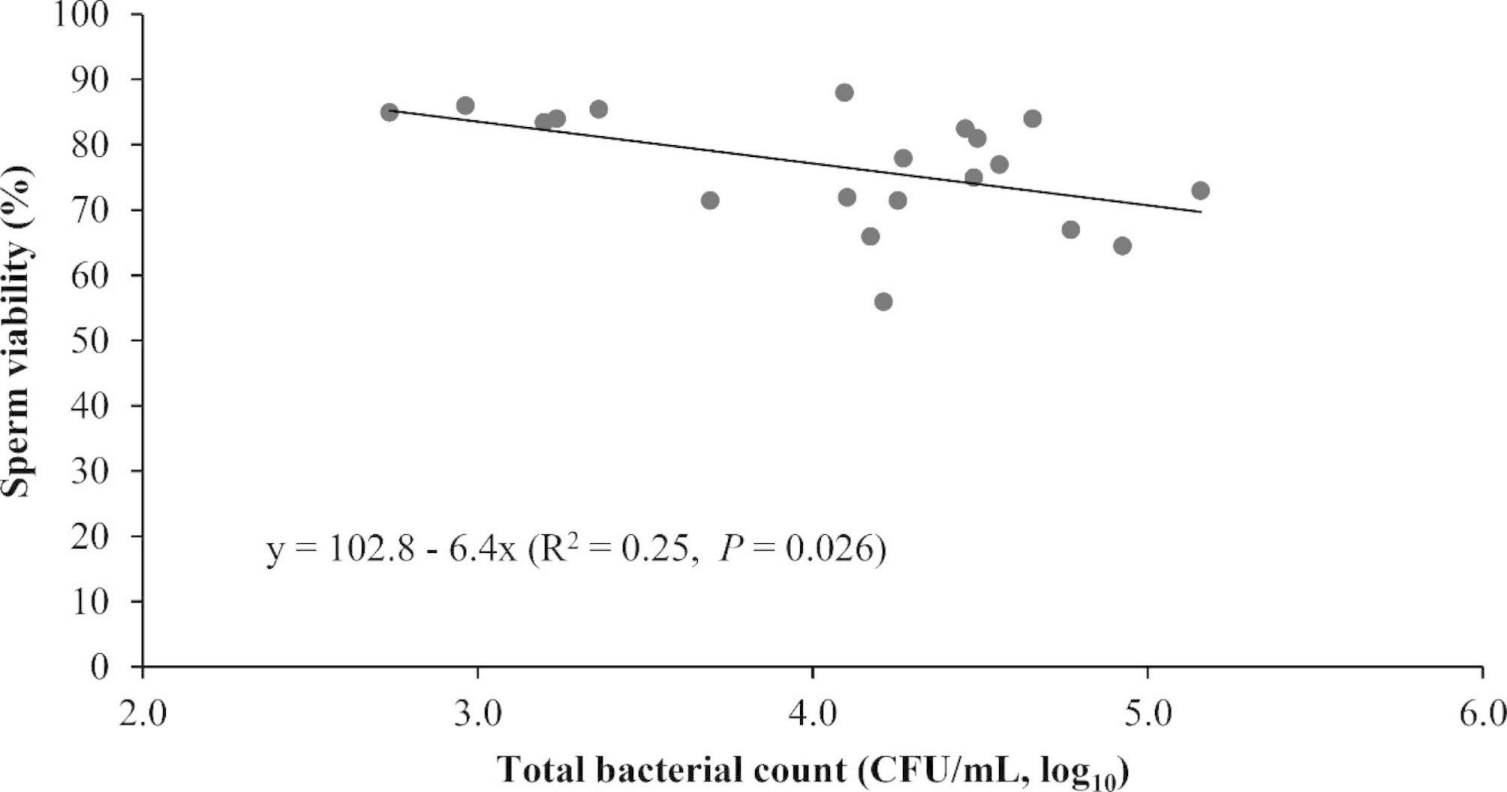
Linear relationship between sperm viability and total bacterial count (CFU/mL, log_10_) in boar semen

Sperm quality parameters and bacterial contamination are presented in Table 1. There was an association between viability and bacterial count (Fig. 1). The total bacterial count varied between ejaculates from 2.7 to 5.2 CFU/mL (log_10_) (Table 1). Interestingly, sperm viability decreased by 6.4% for every extra log_10_ of the total bacteria count. The regression equation could be expressed as sperm viability = 102.8–6.4 × total bacterial count (CFU/mL, log_10_), R^2^ = 0.25; p = 0.026 (Fig. 1). However, the number of *Staphylococcus spp*. and *Proteus spp.* count at levels of 2.4–4.4 CFU/ml (log_10_) and 1.9–4.1 CFU/mL (log_10_), respectively were not associated with sperm viability (*p* > 0.05) or total sperm motility (*p* > 0.05).

**Table 1:** Boar sperm characteristics and contaminants in 20 semen ejaculates

Variables	Means ± SD	Range
Semen volume (mL)	199.3 ± 84.5	62.0–404.0
Sperm motility (%)	72.3 ± 6.4	65.0–85.0
Sperm viability (%)	76.6 ± 8.7	56.0–88.0
Concentration (× 10^6^ sperm/mL)	345.2 ± 104.2	176.0–552.0
Total sperm per ejaculate (× 10^9^ sperm)	65.3 ± 27.7	22.4–124.3
pH	7.5 ± 0.3	7.0–8.0
Number of possible isolated colonies	5.6 ± 1.8	3.0–10.0
Total bacterial count (CFU/mL, log_10_)	4.1 ± 0.7	2.7–5.2
Staphylococcus spp. count (CFU/mL, log_10_)	3.3 ± 0.7	2.1–4.4
Proteus spp. count (CFU/mL, log_10_)	3.0 ± 0.7	1.9–4.1

### Bacteria contamination of fresh semen

**Fig. 2:**
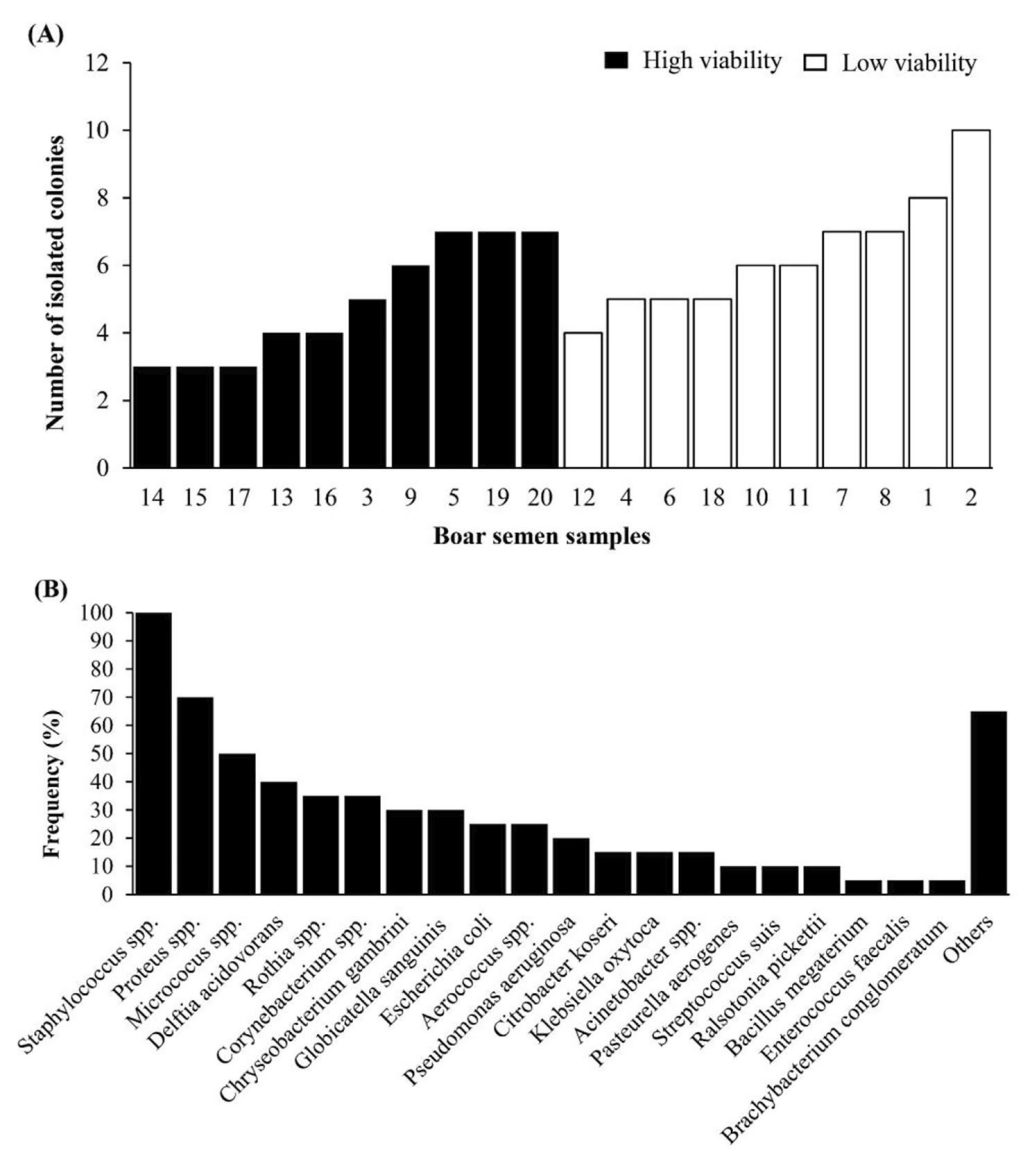
Bacterial contamination in 20 fresh boar semen samples **A.)** Number of isolated colonies from semen samples, classified according to high or low sperm viability; **B.)** Frequency of isolated bacteria from semen samples

**Fig. 3:**
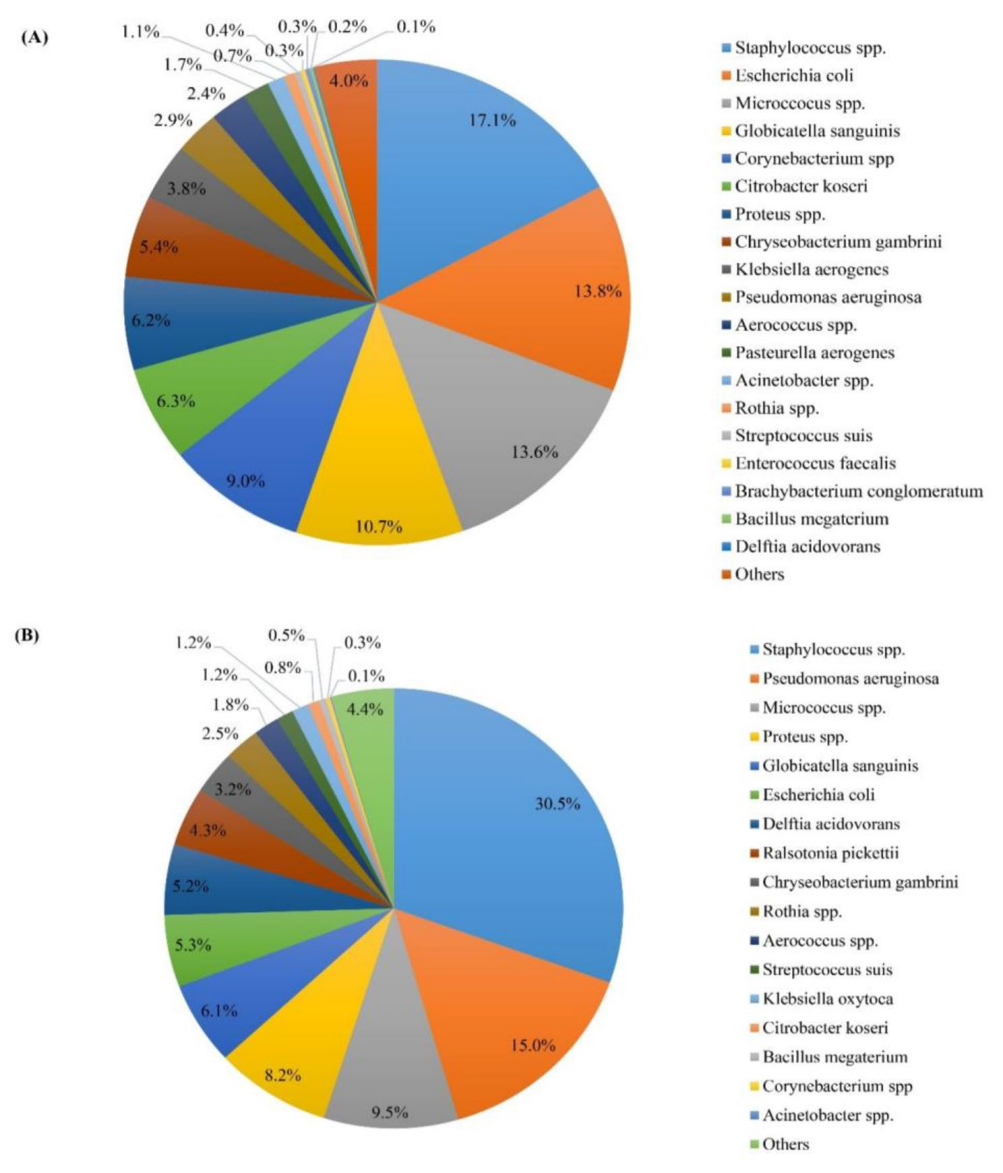
Bacterial composition in **(A)** low-viability semen samples (n = 10) and **(B)** high-viability semen samples (n = 10)

The numbers of isolated colonies and the most frequently isolated bacteria are presented in Fig. 2. The boar ejaculates were contaminated with 3 to 10 types of bacteria (Fig. 2A). *Staphylococcus spp.* (100%), *Proteus spp*. (70%) and *Micrococcus spp.* (50%) were the most frequently isolated genera. In addition, *Bacillus megaterium* (5%), *Enterococcus faecalis* (5%) and *Brachybacterium conglomeratum* (5%) were also detected (Fig. 2B). *Staphylococcus spp.* were the most frequently isolated in either low or high viability boar semen (Fig. 3A and B, respectively).

### Growth of bacteria in semen extender with and without antibiotic

**Fig. 4:**
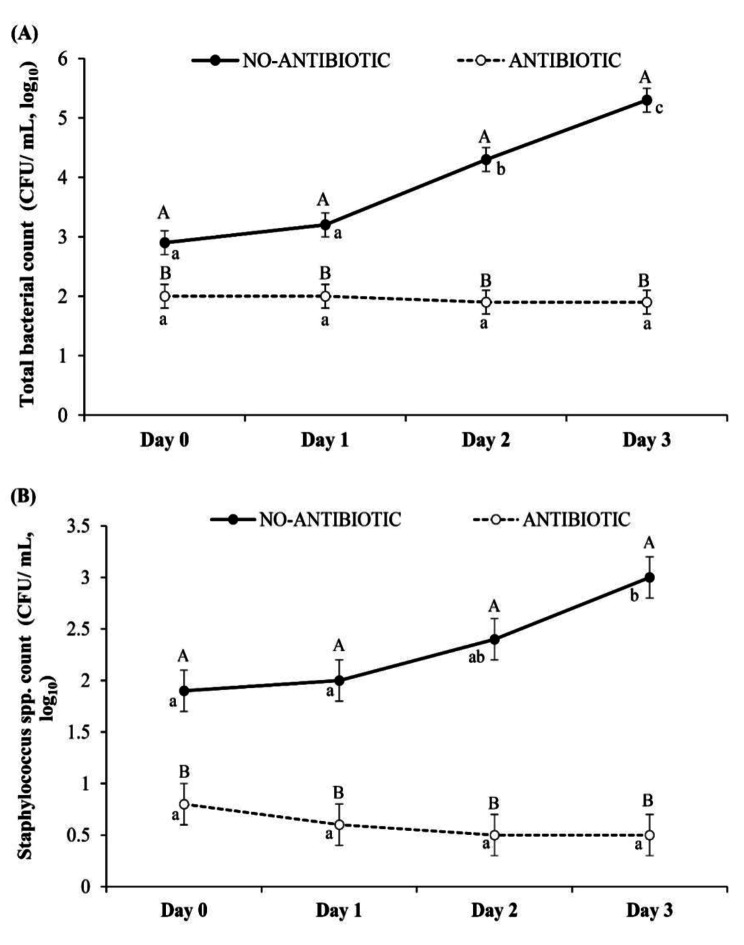
**(A)** Total bacterial count (CFU/mL, log_10_) and **(B)** Staphylococcus spp. count (CFU/mL, log_10_) in semen extender without antibiotic (NO-ANTIBIOTIC) and with antibiotic (ANTIBIOTIC) in extended boar semen preserved for 4 days, ^a,b,c^ Different letters within lines and ^A,B^ different letters between lines indicate significant differences (*p* < 0.05)

On the collection day (day 0), the total number of bacteria counted in fresh semen (4.1 ± 0.2 log_10_) was higher than that in semen diluted in BTS extender without gentamicin (2.9 ± 0.2 log_10_) and semen diluted in BTS extender with gentamicin (2.0 ± 0.2 log_10_) (*p* < 0.001). The differences in the total bacterial counts and *Staphylococcus spp.* counts between NO-ANTIBIOTIC and ANTIBIOTIC during the storage time are presented in Fig. 4. In the NO-ANTIBIOTIC group, the total numbers of bacteria counted on days 2 and 3 of storage were higher than those determined on days 0 and 1 (*p* < 0.001). However, no difference was detected across time points in the ANTIBIOTIC group (*p* > 0.05). Moreover, the total number of bacteria determined in the ANTIBIOTIC group was lower than that in the NO-ANTIBIOTIC group examined on each storage day (*p* < 0.001) (Fig. 4A). Similarly, the total number of *Staphylococcus spp*. counted on day 3 of storage was higher than that on day 0 and day 1 in the NO-ANTIBIOTIC group (*p* < 0.001), but no significant differences were detected in the ANTIBIOTIC group (*p* > 0.05). Also, the growth of *Staphylococcus spp.* in the NO-ANTIBIOTIC group was higher than in the ANTIBIOTIC group on each storage day (p < 0.001) (Fig. 4B).

Interestingly, in the semen with antibiotic, the top three dominant bacteria were *Globicatella sanguinis* (45.4%), *Delftia acidovorans* (17.9%) and *Micrococcus spp.* (7.8%). Moreover, on the last day of preservation, *Globicatella sanguinis (*57.2%), *Delftia acidovorans (18.9%)* and *Micrococcus spp.* (5.9%) remained as the top three most abundant contaminants in the semen with antibiotic.

In the semen without antibiotic, *Staphylococcus spp.* (41.2%), *Proteus spp.* (36.9%) and *Citrobacter koseri* (5.0%) accounted for the most frequently occurring bacterial contaminations during the 4 days of preservation. On the last day of preservation, *Staphylococcus spp.*, *Proteus spp*. and *Citrobacter koseri* were the top three most abundant bacteria, accounting for 42.5%, 34.7%, and 5.1% of the total bacteria, respectively.

### Effects of antibiotic on bacterial contamination

The effects of antibiotic on sperm quality and bacterial contamination in the NO-ANTIBIOTIC and ANTIBIOTIC groups are presented in Tables 2 and 3, respectively. Sperm motility, viability and acrosome integrity in the ANTIBIOTIC group were higher than in the NO-ANTIBIOTIC group (Table 2). The total bacterial count in the ANTIBIOTIC group was lower than in the NO-ANTIBIOTIC group (1.9 ± 0.1 versus 3.9 ± 0.1 log_10_, respectively, *p* < 0.001) (Table 2). Sperm acrosome integrity in the ANTIBIOTIC group was higher than in the NO-ANTIBIOTIC group on days 2 and 3 of preservation. Other sperm parameters did not differ between the two experimental groups (Table 3). The total bacterial count on each storage day in the ANTIBIOTIC group was lower than that in the NO-ANTIBIOTIC group (*p* < 0.001) (Table 3).

**Table 2:** Boar sperm characteristics and bacterial contamination in semen extended in BTS without antibiotic (NO-ANTIBIOTIC) or with antibiotic (ANTIBIOTIC) (least square means ± SEM)

Variables	Group		*p* value
	NO-ANTIBIOTIC	ANTIBIOTIC	
Total sperm motility (%)	65.6 ± 3.4	67.5 ± 3.4	0.023
- Curvilinear velocity (VCL, µm/sec)	83.4 ± 3.5	84.2 ± 3.5	0.204
- Straight-line velocity (VSL, µm/sec)	24.6 ± 1.3	24.8 ± 1.3	0.686
- Average path velocity (VAP, µm/sec)	44.0 ± 2.2	43.7 ± 2.2	0.799
Sperm viability (%)	71.7 ± 2.1	72.8 ± 2.1	0.031
Acrosome integrity (%)	78.5 ± 1.8	79.6 ± 1.8	0.011
Sperm membrane integrity (%)	57.2 ± 2.7	58.2 ± 2.7	0.132
Sperm mitochondrial activity (%)	67.7 ± 2.8	68.5 ± 2.8	0.141
Total bacterial count (CFU/mL, log_10_)	3.9 ± 0.1	1.9 ± 0.1	< 0.001

**Table 3:** Boar sperm characteristics and bacterial contamination in NO-ANTIBIOTIC and ANTIBIOTIC groups on days 1, 2 and 3 of storage (least square means ± SEM)

Variables	Day	Group		*p* value
		NO-ANTIBIOTIC	ANTIBIOTIC	
Total sperm motility (%)	0	72.5 ± 3.6	74.0 ± 3.6	0.370
	1	67.5 ± 3.6	69.1 ± 3.6	0.351
	2	63.7 ± 3.6	65.5 ± 3.6	0.279
	3	58.5 ± 3.6	61.4 ± 3.6	0.094
Sperm viability (%)	0	76.9 ± 2.2	78.2 ± 2.2	0.191
	1	73.0 ± 2.2	74.1 ± 2.2	0.243
	2	69.8 ± 2.2	70.8 ± 2.2	0.298
	3	67.2 ± 2.2	68.0 ± 2.2	0.416
Acrosome integrity (%)	0	82.4 ± 1.8	83.0 ± 1.8	0.406
	1	80.4 ± 1.8	80.7 ± 1.8	0.712
	2	76.6 ± 1.8	78.3 ± 1.8	0.044
	3	74.7 ± 1.8	76.2 ± 1.8	0.054
Sperm membrane integrity (%)	0	65.0 ± 2.8	65.5 ± 2.9	0.701
	1	58.4 ± 2.9	59.6 ± 2.9	0.338
	2	54.2 ± 2.9	55.7 ± 2.9	0.289
	3	51.1 ± 2.9	52.1 ± 2.9	0.472
Sperm mitochondrial activity (%)	0	72.7 ± 2.9	73.6 ± 2.9	0.404
	1	69.8 ± 2.9	70.9 ± 2.9	0.336
	2	66.1 ± 2.9	66.7 ± 2.9	0.607
	3	62.1 ± 2.9	62.9 ± 2.9	0.521
Total bacterial count (CFU/mL, log_10_)	0	2.9 ± 0.2	2.0 ± 0.2	< 0.001
	1	3.2 ± 0.2	2.0 ± 0.2	< 0.001
	2	4.3 ± 0.2	1.9 ± 0.2	< 0.001
	3	5.3 ± 0.2	1.9 ± 0.2	< 0.001

A comparison of sperm quality and the total bacterial counts between the NO-ANTIBIOTIC and ANTIBIOTIC groups, classified as low- and high-viability samples, indicated that sperm mitochondrial activity in the NO-ANTIBIOTIC group was higher than that in the ANTIBIOTIC group (60.2 ± 3.1% versus 58.7 ± 3.1%, respectively, *p* < 0.05). Other sperm parameters did not differ significantly between NO-ANTIBIOTIC and ANTIBIOTIC groups. In high-viability semen samples, all the sperm parameters in the ANTIBIOTIC group were higher than those in the NO-ANTIBIOTIC group (*p* < 0.001). The total bacterial count in the ANTIBIOTIC group was lower than that in the NO-ANTIBIOTIC group (*p* < 0.001) in both low- and high-viability semen samples. Interestingly, in the low-viability semen samples, no differences in any sperm parameters were detected between the NO-ANTIBIOTIC and ANTIBIOTIC groups on each day of storage (*p* > 0.05) (Table 4). However, significant differences were found between the NO-ANTIBIOTIC and ANTIBIOTIC groups on days 2 and 3 in high-viability samples (Table 4). In addition, the total bacterial count in the ANTIBIOTIC group was lower than in the NO-ANTIBIOTIC group on each storage day in both low- and high-viability semen samples (*p* < 0.001) (Table 4).

**Table 4:** Comparison of boar sperm characteristics and bacterial contamination between NO-ANTIBIOTIC and ANTIBIOTIC groups examined on each storage day, in low- and high-viability semen samples (least square means ± SEM).

Variables	Day	Group		*p* value
		NO-ANTIBIOTIC	ANTIBIOTIC	
*Low-viability semen samples*				
Total sperm motility (%)	0	64.0 ± 4.6	65.0 ± 4.6	NS
	1	58.4 ± 4.6	58.9 ± 4.6	NS
	2	54.3 ± 4.6	54.2 ± 4.6	NS
	3	49.1 ± 4.6	49.6 ± 4.6	NS
Sperm viability (%)	0	69.4 ± 2.4	70.7 ± 2.4	NS
	1	66.7 ± 2.4	67.0 ± 2.4	NS
	2	64.0 ± 2.4	63.1 ± 2.4	NS
	3	60.8 ± 3.4	59.4 ± 2.4	NS
Acrosome integrity (%)	0	79.1 ± 2.9	79.6 ± 2.9	NS
	1	77.1 ± 2.9	76.9 ± 2.9	NS
	2	73.2 ± 2.9	73.6 ± 2.9	NS
	3	71.6 ± 2.9	71.9 ± 2.9	NS
Sperm membrane integrity (%)	0	56.5 ± 3.4	56.5 ± 3.4	NS
	1	50.1 ± 3.4	50.8 ± 3.4	NS
	2	46.1 ± 3.4	45.7 ± 3.4	NS
	3	41.4 ± 3.4	40.2 ± 3.4	NS
Sperm mitochondrial activity (%)	0	66.1 ± 3.2	65.6 ± 3.2	NS
	1	62.1 ± 3.2	61.2 ± 3.2	NS
	2	59.3 ± 3.2	56.5 ± 3.2	NS
	3	53.3 ± 3.2	51.1 ± 3.2	NS
Total bacterial count (CFU/mL, log_10_)	0	3.1 ± 0.2a	2.4 ± 0.2b	***
	1	3.5 ± 0.2a	2.4 ± 0.2b	***
	2	4.6 ± 0.2a	2.2 ± 0.2b	***
	3	5.6 ± 0.2a	2.2 ± 0.2b	***
*High-viability semen samples*				
Total sperm motility (%)	0	81.6 ± 2.1	83.5 ± 2.1	NS
	1	77.1 ± 2.1	79.6 ± 2.1	NS
	2	73.7 ± 2.1	77.3 ± 2.1	*
	3	68.6 ± 2.1	73.6 ± 2.1	**
Sperm viability (%)	0	84.8 ± 1.2	86.0 ± 1.2	NS
	1	79.7 ± 1.2	81.7 ± 1.2	NS
	2	76.0 ± 1.2	79.0 ± 1.2	*
	3	74.1 ± 1.2	77.0 ± 1.2	*
Acrosome integrity (%)	0	86.3 ± 1.0	87.1 ± 1.0	NS
	1	84.2 ± 1.0	85.0 ± 1.0	NS
	2	80.7 ± 1.0	83.6 ± 1.0	**
	3	78.4 ± 1.0	81.2 ± 1.0	**
Sperm membrane integrity (%)	0	73.4 ± 1.4	74.3 ± 1.4	NS
	1	66.0 ± 1.4	68.2 ± 1.4	NS
	2	62.3 ± 1.4	65.6 ± 1.4	*
	3	60.6 ± 1.4	63.8 ± 1.4	*
Sperm mitochondrial activity (%)	0	79.2 ± 2.1	81.7 ± 2.1	NS
	1	77.4 ± 2.1	80.2 ± 2.1	NS
	2	73.0 ± 2.1	76.9 ± 2.1	**
	3	71.0 ± 2.1	74.7 ± 2.1	*
Total bacterial count (CFU/mL, log_10_)	0	2.6 ± 0.2	1.7 ± 0.2	***
	1	2.9 ± 0.2	1.5 ± 0.2	***
	2	4.1 ± 0.2	1.7 ± 0.2	***
	3	5.1 ± 0.2	1.5 ± 0.2	***

## Discussion

### Bacteriostatic effect of gentamicin

The present study confirms the hypothesis that the bacteriostatic activity of gentamicin controlled the overgrowth of certain bacterial contaminants in boar semen during short-term storage. The promising effect of gentamicin was apparent on the day of semen collection (day 0), when the total bacterial count in the ANTIBIOTIC group was lower than in the NO-ANTIBIOTIC group. Also, during storage from day 0 to day 3, gentamicin strongly inhibited the total bacterial number. However, the total bacterial count increased dramatically to 5.3 log on the last storage day in the NO-ANTIBIOTIC group. These results are in agreement with Luther et al. [33], who showed that in an environment without gentamicin, the bacterial count increased to 5.6 × 10^6^ CFU/mL but was less than 10^1^ CFU/mL when the BTS extender was supplemented with 0.25 g of gentamicin per liter. In the present study, the bacterial composition and the dominant bacteria differed between the NO-ANTIBIOTIC and ANTIBIOTIC groups. In the NO-ANTIBIOTIC group, 62.5% of the dominant bacteria were Gram-negative and 37.5% were Gram-positive, whereas 60.0% were Gram-positive and 40.0% Gram-negative bacteria in the ANTIBIOTIC group. These findings indicate that the bacteriostatic effect of gentamicin was more effective against Gram-negative than Gram-positive bacteria. This result is in agreement with Gączarzewicz et al. [5], who reported that gentamicin sulphate strongly inhibited Gram-negative bacteria but had a limited activity on Gram-positive contaminants. Moreover, in the present study, the bacterial contaminants on the third day were similar to those during the entire storage period, although the count of each isolated bacterial type dramatically increased. In addition, in the ANTIBIOTIC group, *Globicatella sanguinis, Delftia acidovorans* and *Micrococcus spp.* were the top three most abundant bacteria detected on the last day of the preservation, accounting for 57.2%, 18.9% and 5.9% of the total bacteria, respectively. The increase in bacterial count at the end of storage in the presence of gentamycin indicates the development of antibiotic resistance.

Interestingly, *Staphylococcus spp.* and *Bacillus megaterium* were dominant Gram-positive bacteria in the NO-ANTIBIOTIC group during preservation, accounting for 42.5% and 0.4%, respectively, of the total bacteria. However, the proportion of *Staphylococcus spp.* was 5.2%, and no *Bacillus megaterium* was isolated from the ANTIBIOTIC group. In the case of *Staphylococcus spp.*, gentamicin strongly inhibited Gram-positive bacteria, which were present at a count of 0.5 log instead of 3.0 log measured in the culture without the antibiotic. This could be explained by an acquired resistance of the Gram-positive bacteria to gentamicin. Similarly, Bresciani et al. [34] indicated that in European countries, about 50% of *Staphylococcus epidermidis* showed acquired resistance to gentamicin. Also, in extended porcine semen, some bacteria from the family Enterobacteriaceae showed acquired resistance to aminoglycosides and gentamicin [35]. Consequently, the rational use of gentamicin in semen extenders should be emphasized to avoid undesired effects on other industries. The rational use of gentamicin in boar semen extenders was suggested in the report of Schulze et al. [28], which indicated the importance of investigating the bacterial composition in boar semen and of determining the minimal inhibitory concentration (MIC) range of gentamicin for all isolated bacteria to ensure that the concentration of gentamicin in the semen doses is appropriate.

### The maintenance of semen quality in extender with and without antibiotics

Along with the positive effects of gentamicin in controlling bacterial growth in extended semen, the results also confirm our hypothesis that the current dose of gentamicin can lead to positive results when preserving boar semen, making this approach a preferable solution for rational use of antibiotics in a sustainable livestock breeding industry. Generally, the semen quality in the ANTIBIOTIC group was higher than in the NO-ANTIBIOTIC group in terms of sperm motility, viability and acrosome integrity parameters. Although the total bacterial count in the semen ranged from 10^1^ to 10^6^ CFU/mL (1–6 log_10_), the extended semen was still good for use, with a sperm viability > 65.4% [36] and a mitochondrial activity > 50% [37]. This result is in agreement with Pinart et al. [38], who observed that the threshold values for mesophilic aerobic bacteria could be between 10^3^ and 10^7^ CFU/mL before adverse effects on sperm quality occurred. Furthermore, it was reported that loss of sperm motility, membrane disintegration and sperm agglutination occur at sperm: bacteria ratios of 1:1 or a bacterial count of approximately 2 × 10^7^ CFU/mL [3, 28]. These results could be used as arguments against the requirements of breeding organizations worldwide, namely the absence of bacterial contamination in the semen dose from dilution until the expiration date [13].

Our findings contribute new insights toward reducing antibiotics as well as rational antibiotic used in boar AI industry. The growth of bacteria was significantly different only after 2 days of preservation in the semen without antibiotic. Semen with high viability at the start retained its quality during storage, in contrast to low-viability semen, with or without antibiotics. Regarding the requirements of 11 breeding organizations, the sperm motility on the expiry day must be 45–70%, and bacterial contamination must not exceed 1,000 CFU/mL (~ 2–3 log) when the semen doses are sold on the global market [13]. Also, the threshold values for mesophilic aerobic bacteria should be above 10^7^ CFU/mL to result in adverse effects on sperm quality or fertility [12, 35, 38]. We therefore suggest the following: (i) if ejaculates are collected from boars with high sperm quality, it is possible to omit the use of antibiotics in semen extender and only use the semen doses for up to 2 days, if strict hygienic measures are followed when collecting and processing semen; and (ii) if boar studs sell semen doses on the global market, they can use gentamicin to control bacterial growth to meet the requirements of the breeding organizations as well as to maintain semen quality until day 3. These strategies would be ideal to reduce antibiotic usage for sustainable livestock breeding.

Based on our findings, gentamicin seems to retain the quality of high-viable semen better than lower viable semen. Unlike the high-viable semen, adding antibiotic into the semen extender did not create any benefit on boar sperm characteristics in low viability semen in every storage day compared between NO-ANTIBIOTIC and ANTIBIOTIC groups (Table 4). As a result, adding gentamicin to semen extender would be more valuable when dealing with high-viability semen in terms of maintaining sperm quality and controlling bacterial growth. In addition, there should be more focus on boar selection for good sperm quality, as well as attention to strict hygienic measures during semen collection and processing to reduce bacterial contamination [2].

### Boar seminal bacterial contamination profile

The present study indicates that all ejaculates were contaminated with various bacteria, with at least 20 and 24 different bacterial genera identified from fresh and diluted semen, respectively. The greater number of bacterial genera in the diluted semen samples might be associated with bacterial contamination during semen processing or due to the overgrowth of bacteria in the raw semen, which might have prevented some bacteria from being seen. The number of bacterial contaminations examined in each ejaculate ranged from 3 to 10 colonies. This result is in agreement with a number of previous studies [5, 6, 39]. It has been demonstrated that almost all ejaculates used in scientific research were contaminated with 2 or 3 types of bacteria, and at least 25 different bacterial types were isolated [5, 6, 39]. Althouse et al. [3] reported that 10 to 15 colonies were recovered from extended semen samples. The similarity in the results of the present study and other cited reports emphasizes that bacterial contamination in boar semen is universal. Furthermore, it confirms the limitations of the current bacterial culture and identification techniques such as gram staining, biochemical tests and MALDI-TOF in investigating the number of contaminants. Moreover, the difference in the number of isolated bacteria between fresh and diluted semen in the present study confirmed that the source of bacterial contamination in boar semen is not only derived from the boars themselves but may also come from the environment [13]. However, bacteria coming from the boar and from the environment cannot be clearly differentiated. Thus, any effort to eliminate or inhibit the growth of bacteria in boar semen must follow strict hygienic measures applied during semen collection and subsequent laboratory processing [27, 33].

Of the 24 bacterial genera isolated from the diluted semen, 13 (54.2%) were Gram-negative; most of them belonged to the Enterobacteriaceae family, followed by Pseudomonadaceae, Pasteurellaceae and Comamonadaceae. The remaining 11 (45.8%) bacteria were Gram-positive, such as *Staphylococcus spp.*, *Streptococcus spp*. and *Globicatella sangunis*, belonging to different families (Staphylococcaceae, Streptococcaceae and Aerococcaceae, respectively). These results confirmed a previous study [5] in which 6 species of Gram-negative bacteria isolated from 79 ejaculates belonged to the Enterobacteriaceae and Pseudomonadaceae families, and 3 species of Gram-positive bacteria, including *Staphylococcus spp.*, *Streptococcus spp*. and *Bacillus spp*., were identified. Ubeda et al. [35] also stated that most bacteria contaminating boar semen were members of the family Enterobacteriaceae, such as *Proteus mirabilis*, *Klebsiella oxytoca* and *Monganella morganii*. Also, a large number of contaminants isolated from extended semen were gram-negative bacteria from the family Enterobacteriaceae [3, 39].

In the present study, *Staphylococcus spp*., *Proteus spp*. and *Micrococcus spp.* were frequently detected in 100%, 70% and 50% of the 20 fresh ejaculates, respectively. The isolated bacteria appearing with lower frequencies included *Bacillus megatherium* (5%), *Enterococcus faecalis* (5%) and *Brachybacterium conglomeratum* (5%). There was a difference in the genera of bacteria most frequently identified compared to other studies. For instance, Sone [40] found that *Pseudomonas* was the most frequently isolated bacterial genus, along with *Enterobacter cloacae*, *Escherichia coli* and *Serratia marcescens* [3]. However, in a different study, *Staphylococcus spp*. was the most frequently isolated genus [5]. In the present study, the most frequently isolated bacteria were *Staphylococcus* spp. This can be explained by the differences among boar studs, bacterial exposure and hygienic measures as well as laboratory techniques, which could result in variation in the frequency of isolated bacteria.

To confirm our hypothesis, dilution of the ejaculates reduced the total bacterial count in fresh ejaculates from 4.1 log to 2.9 log. This result was in agreement with Gączarzewicz et al. [5], who reported that the average total number of bacteria in native semen was reduced from 724 × 10^3^ to 2.7 × 10^3^ CFU/mL by dilution. In this study, the number of bacteria in fresh semen reflected the poor hygiene practices in the boar stud and the different properties of the boars. Goldberg et al. [41] found that the median value of boar seminal contaminants was 220 CFU/mL, which could be increased under less hygienic conditions. Our results are consistent with Schulze [28], who reported bacterial counts in boar semen ejaculates from 0 to 10^5^ CFU/mL [42].

### Association between bacteriospermia and sperm quality

The present study confirms a number of previous studies that bacteriospermia compromise boar sperm quality [5, 6, 10, 12]. In the present study, we demonstrated that sperm viability decreased by 6.4% for every extra log_10_ of the total bacteria count. However, *Staphylococcus spp.* and *Proteus spp.* at the levels of 3.3 log_10_ and 3.0 log_10_, respectively, did not cause undesired effects on viability and motility of boar sperm. These results indicate that the effect of the contaminants on sperm quality is not only dependent on the genera but also on the number of bacteria. Clearly, the level of 4.1 log represents a high contamination load, with a loss of sperm viability, although the latter was not caused by *Staphylococcus spp.* and *Proteus spp.*. Consequently, bacterial investigation must be performed to identify the bacteria with detrimental effects on sperm quality. This finding is in agreement with Sone [40], who revealed that even at 10 to 12 CFU/mL log levels, *Staphylococcus spp.* caused a moderate reduction in pH (6.3–6.5) but almost no detrimental effects on boar sperm quality. However, *Pseudomonas aeruginosa* and *Clostridium perfringens* caused a decrease in sperm motility, viability and acrosome integrity at the 7 to 8 CFU/mL log thresholds [43, 44]. Moreover, the present study confirmed that bacterial contamination is associated with sperm quality, and an increase in the total colony forming units per mL in fresh semen was associated with undesired effects on sperm viability [5]. This association indicates that sperm viability could be affected by the diversity of the native bacterial flora in the boar reproductive tract as well as the host immune system before semen collection and processing. Although *Staphylococcus spp.* was the dominant type, it had no significant effect on sperm quality. However, sperm quality could be affected by other, less frequently isolated, bacteria. Schulze et al. [28] reported that from 334 samples, 5.7% of *Pseudomonas aeruginosa* in the seminal microbiota was associated with reduced sperm motility, viability and acrosome integrity, and 6.4% of *Escherichia coli* in 250 samples was associated with damaged acrosomes and poor viability [39].

## Conclusion

Sperm viability decreased by 6.4% for every extra log_10_ of the total bacteria count. Along with positive effects on inhibiting bacterial growth during storage, gentamicin retains the quality of high-viability semen better than lower-viability semen. Our findings contribute new insights toward reducing antibiotics as well as rational antibiotic use in the boar AI industry. The growth of bacteria was significantly different only after 2 days of preservation in semen without antibiotic. For semen doses diluted from highly viable ejaculates, it is possible to storage within 2 days without antibiotic supplementation. Interestingly, counts of *Globicatella sanguinis*, *Delftia acidovorans* and *Micrococcus spp.*, that were the top three most abundant contaminants in the boar semen, increased at the end of storage in the presence of gentamicin, suggesting the loss of bacteriostatic properties of gentamicin to the growth of bacteria during storage.

## Data Availability

The datasets used and/or analyzed during the current study are available from the corresponding author upon reasonable request.

## Abbreviations

AI: Artificial insemination
BTS: Beltsville Thawing Solution
CASA: Computer-assisted sperm analysis system
